# Impact of microRNA polymorphisms on high-dose methotrexate-related hematological toxicities in pediatric acute lymphoblastic leukemia

**DOI:** 10.3389/fped.2023.1153767

**Published:** 2023-06-13

**Authors:** Min Zhan, Ting Liu, Zhou Zhang, Guoqiang Wang, Zhongqiang Cao, Xuejuan Li, Hongwu Zeng, Huirong Mai, Zebin Chen

**Affiliations:** ^1^ Department of Pharmacy, Shenzhen Children’s Hospital, Shenzhen, China; ^2^ Department of Radiology, Shenzhen Children’s Hospital, Shenzhen, China; ^3^ Department of Hematology/Oncology, Shenzhen Children’s Hospital, Shenzhen, China

**Keywords:** acute lymphoblastic leukemia, pediatric, miRNA polymorphism, methotrexate, hematological toxicities

## Abstract

**Objectives:**

It is well known that transporter and enzyme genes could be regulated by microRNA (miRNA) at the post-transcriptional level, and single-nucleotide polymorphisms (SNPs) in miRNA, which are involved in the miRNA production and structure, may impact the miRNA expression level and then influence drug transport and metabolism. In this study, we aim to evaluate the association between miRNA polymorphisms and high-dose methotrexate (HD-MTX) hematological toxicities in Chinese pediatric patients with acute lymphoblastic leukemia (ALL).

**Method:**

A total of 181 children with ALL were administered with 654 evaluable cycles of HD-MTX. Their hematological toxicities were evaluated according to the National Cancer Institute Common Terminology Criteria for Adverse Events v5. The association between 15 candidate SNPs of miRNA and hematological toxicities (leukopenia, anemia, and thrombocytopenia) was analyzed using Fisher's exact test. Further multiple backward logistic regression analysis was used to explore the independent risk factors for grade 3/4 hematological toxicities.

**Result:**

Rs2114358 G>A in pre-hsa-miR-1206 was related to HD-MTX-related grade 3/4 leukopenia after multiple logistic regression [GA + AA vs. GG: odds ratio (OR): 2.308, 95% CI: 1.219–4.372, *P* = 0.010], and rs56103835 T > C in pre-hsa-mir-323b was associated with HD-MTX-related grade 3/4 anemia (TT + TC vs. CC: OR: 0.360, 95% CI: 0.239–0.541, *P* = 0.000); none of the SNPs were significantly associated with grade 3/4 thrombocytopenia. Bioinformatics tools predicted that rs2114358 G>A and rs56103835 T>C would impact the secondary structure of pre-miR-1206 and pre-miR-323b, respectively, and then probably influence the expression level of mature miRNAs and their target genes.

**Conclusion:**

Rs2114358 G>A and rs56103835 T>C polymorphism may potentially influence HD-MTX-related hematological toxicities, which may serve as candidate clinical biomarkers to predict grade 3/4 hematological toxicities in pediatric patients with ALL.

## 1. Introduction

Methotrexate (MTX), a folic acid antagonist, is widely used in therapy for leukemia, lymphoma, osteosarcoma, and immune disease (1, 2). However, due to the poor selectivity of MTX, it acts not only on tumor cells but also on normal cells, such as hematopoietic stem cells, intestinal epithelial cells, and oral mucosal cells, with vigorous proliferation, leading to bone marrow suppression, gastrointestinal reactions, and oral mucositis (3–5). These toxicities may result in dose reduction, treatment failure, or even death. In addition, patients with severe toxicity will likely experience prolonged hospital stays, delayed chemotherapy, and increased economic burden (3, 6–8).

MicroRNA (miRNA) is a short non-coding RNA [∼22 nucleotides (nt)] that regulates gene expression at the post-transcriptional level through binding to the 3'-untranslated region of specific mRNAs, leading to mRNA degradation or translational inhibition (9). The biogenesis of miRNAs is a multiple-step biological process. miRNA genes are transcribed to produce the long primary transcripts (pri-miRNAs) in the nucleus. The pri-miRNAs are processed to generate stem-loop precursors of 70–90 nt (pre-miRNAs). The pre-miRNAs are then exported to the cytoplasm, where they are cleaved by DICER to generate mature miRNAs (∼22 nt) (10, 11).

Single-nucleotide polymorphisms (SNPs) are the most common form of genomic variation and contribute substantially to human phenotypic differences (12). Increasing evidence demonstrated that SNPs in pre-miRNA and mature miRNA may potentially alter various biological processes by influencing the target selection of miRNAs or mature miRNA abundances (13–16). With regard to the role of miRNA SNP in MTX treatment, rs56103835 in miR-323b, out of 46 SNPs in 42 pre-miRNAs, was significantly associated with MTX plasma clearance, possibly through targeting MTX transporter *ABCC4* (17). Another study revealed that rs56292801 in miR-5189, rs4909237 in miR-595, and rs78790512 in miR-6083 were associated with MTX plasma levels. These miRNAs were predicted to regulate genes involved in MTX uptake transporters: *SLC46A1*, *SLC19A1*, and *SLCO1A2* (18). Furthermore, SNPs in the miRNA were involved in the hepatotoxicity in children with acute lymphoblastic leukemia (ALL) in the Spanish population (19).

In Chinese patients, Wang et al. have reported that miRNA binding site polymorphism of *MTHFR*, *SLC19A1*, and *SLCOA2* influenced MTX concentration in ALL patients (20–23). However, no study evaluated the association between miRNA SNPs and HD-MTX-related toxicities in Chinese pediatric patients. Meanwhile, myelosuppression occurred in ∼50% of patients who received HD-MTX administration.

Therefore, the objective of this study is to explore the relationship between miRNA SNPs and HD-MTX-related hematological toxicities (leukopenia, anemia, and thrombocytopenia) in Chinese pediatric patients with ALL.

## 2. Materials and methods

### 2.1. Patients

This retrospective study enrolled a total of 181 pediatric patients diagnosed with ALL who received treatment at Shenzhen Children's Hospital in Guangdong, China, from December 2015 to April 2019. All patients detected with at least one serum MTX concentration at 48 or 72 h after MTX infusion and whose DNA samples could be obtained were included.

Demographic and clinical information of the patients was obtained from their medical records, encompassing age, sex, weight, treatment regimen, dose, diagnosis, and risk group. In addition, laboratory test results were collected, including white blood cell (WBC), red blood cell (RBC), platelet (PLT), alanine aminotransferase (ALT), total protein (TP), total bilirubin (TBIL), and creatinine (Cr). All laboratory values were measured at different time points within the 7 days preceding the administration of high-dose MTX (HD-MTX); however, only the results closest to the MTX administration were utilized for this study's analysis.

Approval from the Ethics Committee of Shenzhen Children's Hospital was obtained. Written informed consent was obtained from all participants in accordance with the Declaration of Helsinki.

### 2.2. Treatment protocol

The ALL patients were treated with either GD-2008-ALL or SCCLG-2016-ALL protocol (24). During the consolidation phase, all involved patients received HD-MTX treatment at 2 or 5 g/m^2^ based on their body surface area. The first dose (one-tenth of the total dose; no more than 500 mg) was administered as a bolus within 30 min, and the remaining dose was infused over the next 23.5 h.

All patients received hydration and alkalization 12 h before the start of MTX infusion, which continued until the end of rescue according to the protocols. Leucovorin rescue (15 g/m^2^) was started 48 h after HD-MTX infusion and continued for at least every 6 h until the MTX level was less than 0.2 μmol/L.

### 2.3. Determination of serum MTX level

The serum MTX concentration was detected by a polarization fluorescence immunoassay (Siemens) at 24, 48, and 72 h after MTX infusion. Measurement was made daily until the concentration was below 0.2 μmol/L.

### 2.4. SNP selection and genotype determination

SNPs were selected according to the previous association analysis between miRNA SNPs and concentration/toxicity of HD-MTX with minor allele frequency (≥0.05) in the Chinese Han population (17, 18, 25, 26).

Peripheral blood samples were collected from the leftover MTX monitoring samples and were stored at −80°C. DNA was extracted using a blood DNA kit (Qiagen). DNA concentration was qualified by NanoDrop 2000 (Thermo Scientific, Waltham, MA, United States). The genotype of 15 selected SNPs was detected by using the MassARRAY method (Agena Bioscience, San Diego, CA, United States).

### 2.5. Toxicity evaluation

Toxicity data were collected from the medical records of the patients blinded to genotypes. To quantify the degree of myelosuppression, the nadir hemoglobin, PLT, and white cell counts were collected within 14 days after the HD-MTX administration. Toxicity was graded according to the National Cancer Institute Common Terminology Criteria for Adverse Events v5 (27).

### 2.6. Statistical analysis

SNPs were excluded while genotyping call rates less than 95%. The remaining SNPs were tested for Hardy–Weinberg equilibrium (HWE) using the *χ*^2^ test. The SNPs which deviated from HWE were excluded from the analysis.

Continuous variables were interpreted as mean ± SD, while classified variables were presented as numbers or percentages. The relationships between SNPs and hematological toxicities of HD-MTX were analyzed using Fisher's exact test or the *χ*^2^ test. The procedure of Benjamini–Hochberg false discovery rate (FDR) was used to control the error of multiple comparisons, yielding *P*_fdr_.

The association between categorical variables and hematological toxicities was evaluated by the *χ*^2^ test or Fisher's exact test. The relationship between numeric variables and hematological toxicities was evaluated using ANOVA or the Mann–Whitney–Wilcoxon test.

In our study, we analyzed the impact of SNPs and other clinical factors on hematologic toxicity in each treatment cycle. Our study included 181 patients, with a total of 654 observation cycles, resulting in an average of approximately 3.61 cycles per patient, creating an issue of artificially increasing the number of observations. Therefore, in our study, we used a significance level of *P* < 0.05 to determine statistical significance. However, when conducting multivariate analysis, we used a lower significance level (*P* < 0.01) as a criterion for the inclusion of the factors found associated with the toxicities in a univariate analysis into the multivariate analysis. In addition, multivariable backward logistic regression was used to analyze whether miRNA SNPs were associated with grade 3/4 hematological toxicities as a dichotomous variable (yes or no). Patients with grade 3 or 4 toxicities (leukopenia, anemia, and thrombocytopenia) were considered toxic. The backward logistic regression method starts by including all variables in the model. It then gradually eliminates variables that are not statistically significant until only variables with a significant effect on the outcome remain.

### 2.7. *In silico* analysis

The RNAfold web tool (http://rna.tbi.univie.ac.at/cgi-bin/RNAWebSuite/RNAfold.cgi) was used to explore the *in silico* effect of the SNPs on the miRNA structure (28). This web tool calculates the minimum free energy of secondary structures and the energy change (ΔΔ*G*) of the hairpin structure of the miRNAs.

### 2.8. Target prediction

The putative human target miRNA genes were analyzed using TargetScan (version 6.0; targetscan.org/). The Pharmacogenomic Knowledge Base (PharmGKB) (https://www.pharmgkb.org/) was used to identify genes involved in the MTX pathway out of the total target genes.

## 3. Results

### 3.1. Demographic data of patients and MTX-related hematological toxicity

This study included 181 pediatric patients, 1.06–14.10 years old (median 4.24 years old), treated in the Department of Oncology and Hematology, Shenzhen Children's Hospital, in accordance with GD-2008 (36 patients) and SCCLG-2016 (145 patients) protocols. A total of 103 males (56.91%) and 78 females (43.09%) were among the 181 cases (Table 1). The pathological classification included 173 cases (68.3%) of B cell acute lymphoblastic leukemia and 8 cases (14.3%) of T cell acute lymphoblastic leukemia.

**Table 1:** The basic characteristics of the patients.

Patient characteristics	Value
Total number of patients	181
Sex, *n* (%)	
Male	103 (56.91%)
Female	78 (43.09%)
Weight (kg)	19.16 ± 8.44
Age (year)	5.08 ± 2.82
Protocol, *n* (%)	
GD-2008	36 (19.89%)
SCCLG-2016	145 (80.11%)
Risk classification, *n* (%)	
Low risk	36 (19.89%)
Intermediate risk	103 (56.91%)
High risk	42 (23.20%)
Diagnosis	
B-ALL	173 (95.58%)
T-ALL	8 (4.42%)

B-ALL, B cell acute lymphoblastic leukemia; T-ALL, T cell acute lymphoblastic leukemia; *n*, number.

Together, 654 MTX–chemotherapy evaluable cycles administered in the consolidation phase were studied. MTX-related leukopenia was noted in 98.01% (641/654) cycles and was mainly of grade 3/4 (74.31%, 486/654). Further, 96.48% of the cycles had anemia, 26.45% of which were graded 3/4 (Table 2).

**Table 2:** HD-MTX-related hematological toxicities by cycle number.

Toxicity	Grade 0	Grade 1	Grade 2	Grade 3	Grade 4	Grade 3/4
Leukopenia	13 (1.99%)	47 (7.19%)	108 (16.51%)	172 (26.30%)	314 (48.01%)	486 (74.31%)
Anemia	23 (3.52%)	226 (34.56%)	232 (35.47%)	162 (24.77%)	11 (1.68%)	173 (26.45%)
Thrombocytopenia	460 (70.34%)	37 (5.66%)	29 (4.43%)	41 (6.27%)	87 (13.30%)	128 (19.57%)

HD-MTX, high-dose methotrexate.

### 3.2. Association between miRNA polymorphisms and HD-MTX-related hematological toxicities

A total of 15 SNPs in 15 miRNA coding genes were detected in our study (details in Supplementary Table S1). However, Rs11055070 was excluded from the analysis because all the subjects are of the same genotype (TT), and rs8078913 was excluded because the call rate was less than 95%. The remaining 13 SNPs were consistent with the HWE and were included in the next analysis.

To explore if miRNA SNPs may influence hematological toxicity, we tested the association between the 13 SNPs in miRNA and leukopenia, anemia, and thrombocytopenia. The results demonstrated that rs2114358 was related to leukopenia (Table 3). Furthermore, according to the significance level of *P* < 0.01, rs10505168 and rs56103835 are associated with anemia (Table 3). Supplementary Tables S2–S4 demonstrate the detailed results of the association between SNPs and hematological toxicities.

**Table 3:** The association between SNP and hematological toxicity.

SNP	Leukopenia		Anemia		Thrombocytopenia	
	*P* ^a^	*P*_fdr_ ^b^	*P* ^a^	*P*_fdr_ ^b^	*P* ^a^	*P*_fdr_ ^b^
rs10505168	0.068	0.295	0.001	0.007	0.090	0.195
rs1572687	0.344	0.603	0.702	0.702	0.031	0.081
rs2114358	0.000	0.000	0.416	0.486	0.504	0.596
rs2368392	0.156	0.457	0.015	0.0498	0.211	0.305
rs243080	0.890	0.890	0.137	0.254	0.418	0.543
rs35613341	0.458	0.603	0.021	0.0498	0.023	0.075
rs4674470	0.211	0.457	0.226	0.335	0.146	0.271
rs4909237	0.181	0.457	0.449	0.486	0.669	0.669
rs56103835	0.633	0.686	0.000	0.000	0.000	0.000
rs56292801	0.510	0.603	0.017	0.0498	0.021	0.075
rs60871950	0.011	0.072	0.263	0.342	0.014	0.075
rs62571442	0.464	0.603	0.023	0.0498	0.199	0.305
rs78790512	0.376	0.603	0.232	0.335	0.622	0.669

SNP, single-nucleotide polymorphism.

^a^ Fisher's exact test.

^b^ Benjamini–Hochberg.

### 3.3. Clinical factors significantly associated with HD-MTX-related hematological toxicities

We further considered other possible clinical factors that affected MTX-related hematological toxicities. Univariate analysis was used to test the association between the clinical factors and hematological toxicities (leukopenia, anemia, and thrombocytopenia).

As shown in Table 4, leukopenia is likely to be influenced by WBC, RBC, PLT, ALT ratio, TP, dose, diagnosis, and risk. Both anemia and thrombocytopenia are likely to be influenced by age, weight, dose, protocol, diagnosis, risk, WBC, RBC, PLT, ALT ratio, TBIL, TP, Cr ratio, and MTX concentration 48 h after the start of the infusion (C48h). Supplementary Tables S5–S7 demonstrate the detailed results of the association between clinical factors and hematological toxicities.

**Table 4:** Clinical factors associated with MTX-related hematological toxicity.

Variable	Leukopenia^c^	Anemia^c^	Thrombocytopenia^c^
Age	0.409	0.022	0.005
Weight	0.708	0.017	0.030
WBC	0.000	0.000	0.003
RBC	0.000	0.000	0.019
PLT	0.000	0.000	0.000
ALT ratio^a^	0.000	0.000	0.000
TBIL	0.372	0.000	0.000
TP	0.000	0.000	0.000
Cr ratio^b^	0.470	0.001	0.015
C48h	0.079	0.025	0.002
C72h	0.073	0.267	0.066
Sex	0.192	0.239	0.052
Dose	0.000	0.000	0.000
Protocol	0.255	0.015	0.007
Diagnosis	0.043	0.000	0.000
Risk	0.000	0.000	0.000

WBC, white blood cell; RBC, red blood cell; PLT, platelet; ALT, alanine aminotransferase; TBIL, total bilirubin; TP, total protein; Cr, creatinine; C48h, the MTX concentration of 48 h after the start of the infusion; C72h, the MTX concentration of 72 h after the start of the infusion; MTX, methotrexate.

^a^ ALT ratio, ALT/upper limit of reference range.

^b^ Creatinine ratio, creatinine/upper limit of reference range.

^c^ Categorical variables, *χ*^2^-test or Fisher's exact test; numeric variables, ANOVA or Mann–Whitney–Wilcoxon test.

### 3.4. Multivariate backward logistic analysis identified risk factors for grade 3/4 hematological toxicities

Based on the univariate analysis, SNPs and clinical factors with *P* < 0.01 were included in multiple backward binary logistic regression to identify the independent risk factors for the occurrence of grade 3/4 hematological toxicities before every HD-MTX course.

For leukopenia, rs2114358, WBC, RBC, PLT, ALT ratio, TP, dose, and risk were included in the multiple backward logistic regression. For anemia, rs10505168, rs56103835, WBC, RBC, PLT, ALT ratio, TBIL, TP, Cr ratio, dose, diagnosis, and risk were included. For thrombocytopenia, rs56103835, age, WBC, PLT, ALT ratio, TBIL, TP, C48h, dose, protocol, diagnosis, and risk were included in the multiple backward logistic regression.

As shown in Table 5 (step 2), after backward logistic regression, the predictive factors for the occurrence of the MTX-related grade 3/4 leukopenia were WBC [odds ratio (OR): 0.786, 95% CI: 0.690–0.894, *P* = 0.000], RBC (OR: 0.247, 95% CI: 0.151–0.393, *P* = 0.000), ALT ratio (OR: 1.507, 95% CI: 1.178–1.984, *P* = 0.002), risk-HR (OR: 65.823, 95% CI: 16.323–21.909, *P* = 0.000), and rs2114358 GA+AA (OR: 2.308, 95% CI: 1.129–4.372, *P* = 0.010).

**Table 5:** Multiple logistic regression for predicting the risk of grade 3/4 leukopenia.

	Estimate	Std. error	Pr (>|*z*|)	OR	95% CI	
					Lower	Upper
Step 1						
WBC	−0.242	0.065	0.000***	0.785	0.687	0.894
RBC	−1.308	0.266	0.000***	0.270	0.158	0.451
PLT	−0.001	0.001	0.462	0.999	0.998	1.001
ALT ratio^a^	0.387	0.135	0.004**	1.472	1.146	1.945
TP	0.021	0.026	0.423	1.021	0.970	1.075
Dose	0.099	0.108	0.359	1.104	0.892	1.362
Risk-HR	4.018	0.939	0.000***	55.601	11.173	509.527
Risk-IR	−0.176	0.330	0.593	0.838	0.439	1.602
rs2114358GA+AA	0.775	0.328	0.018*	2.171	1.138	4.145
Step 2						
WBC	−0.241	0.064	0.000***	0.786	0.690	0.894
RBC	−1.399	0.243	0.000***	0.247	0.151	0.393
ALT ratio^a^	0.410	0.132	0.002**	1.507	1.178	1.984
Risk-HR	4.187	0.849	0.000***	65.823	16.323	21.909
Risk-IR	0.040	0.238	0.868	1.040	0.649	1.654
rs2114358GA+AA	0.836	0.324	0.010**	2.308	1.219	4.372

WBC, white blood cell; RBC, red blood cell; PLT, platelet; ALT, alanine aminotransferase; TP, total protein; HR, high risk; IR, intermediate risk; OR, odds ratio; 95% CI, confidential interval.

^a^ ALT ratio, ALT/upper limit of reference range.

* *P* < 0.05.

** *P* < 0.01.

*** *P* < 0.001.

As shown in Table 6 (step 3), after backward logistic regression, the independent risk factors for MTX-related grade 3/4 anemia included ALT ratio (OR: 1.367, 95% CI: 1.144–1.641, *P* = 0.001), TBIL (OR: 1.114, 95% CI: 1.062–1.172, *P* = 0.000), RBC (OR: 0.435, 95% CI: 0.276–0.678, *P* = 0.000), dose (OR: 2.359, 95% CI: 1.829–3.203, *P* = 0.000), and rs56103835 TT+TC (OR: 0.360, 95% CI: 0.239–0.541, *P* = 0.000).

**Table 6:** Multiple logistic regression for predicting the risk of grade 3/4 anemia.

	Estimate	Std. error	Pr (>|*z*|)	OR	95% CI	
					Lower	Upper
Step 1						
WBC	0.037	0.073	0.614	1.037	0.906	1.203
RBC	−1.669	0.309	0.000***	0.188	0.101	0.340
PLT	−0.002	0.001	0.026*	0.998	0.995	1.000
ALT ratio^a^	0.447	0.113	0.000***	1.563	1.256	1.959
TBIL	0.079	0.033	0.016*	1.082	1.017	1.155
TP	−0.025	0.026	0.325	0.975	0.927	1.025
Cr ratio^b^	1.144	0.686	0.095	3.139	0.748	11.749
Dose	0.631	0.319	0.048*	1.880	1.105	4.004
Diagnose-T-ALL	−0.346	0.527	0.512	0.708	0.254	2.029
Risk-HR	2.049	1.056	0.052	7.760	0.741	52.451
Risk-IR	−1.351	1.013	0.183	0.259	0.026	1.554
rs10505168CT	−0.981	0.326	0.003**	0.375	0.196	0.708
rs10505168T	−0.957	0.346	0.006**	0.384	0.193	0.751
rs56103835TT+TC	−0.532	0.271	0.049*	0.587	0.345	1.000
Step 2						
RBC	−0.813	0.237	0.001***	0.443	0.276	0.702
PLT	0.000	0.001	0.979	1.000	0.998	1.002
ALT ratio^a^	0.311	0.092	0.001***	1.365	1.142	1.640
TBIL	0.106	0.026	0.000***	1.112	1.059	1.171
Dose	0.852	0.142	0.000***	2.344	1.816	3.186
rs10505168CT	−0.298	0.266	0.263	0.743	0.441	1.253
rs10505168T	−0.312	0.279	0.264	0.732	0.423	1.265
rs56103835TT+TC	−1.065	0.217	0.000***	0.345	0.224	0.526
Step 3						
ALT ratio^a^	0.312	0.092	0.001***	1.367	1.144	1.641
TBIL	0.108	0.025	0.000***	1.114	1.062	1.172
RBC	−0.832	0.229	0.000***	0.435	0.276	0.678
Dose	0.858	0.141	0.000***	2.359	1.829	3.203
rs56103835TT+TC	−1.020	0.209	0.000***	0.360	0.239	0.541

WBC, white blood cell; RBC, red blood cell; PLT, platelet; ALT, alanine aminotransferase; TBIL, total bilirubin; TP, total protein; Cr, creatinine; T-ALL, T cell acute lymphoblastic leukemia; HR, high risk; IR, intermediate risk; OR, odds ratio; 95% CI, confidential interval.

^a^ ALT ratio, ALT/upper limit of reference range.

^b^ Creatinine ratio, creatinine/upper limit of reference range.

*** *P* < 0.001.

With regard to the occurrence of grade 3/4 thrombocytopenia (Table 7 step 2), significant associations were found with PLT (OR: 0.995, 95% CI: 0.993–0.997, *P* = 0.000) and TP (OR: 0.918, 95% CI: 0.883–0.954, *P* = 0.000).

**Table 7:** Multiple logistic regression for predicting risk of grade 3/4 thrombocytopenia.

	Estimate	Std. error	Pr (>|*z*|)	OR	95% CI	
					Lower	Upper
Step 1						
Age	0.000	0.000	0.078	1.000	1.000	1.001
WBC	−0.112	0.063	0.077	0.894	0.781	1.011
PLT	−0.007	0.002	0.000***	0.993	0.990	0.996
ALT ratio^a^	0.209	0.128	0.101	1.233	0.959	1.586
TBIL	0.037	0.037	0.312	1.038	0.969	1.118
TP	0.095	0.029	0.001***	1.099	1.040	1.165
C48h	0.084	0.045	0.065	1.087	0.995	1.206
Dose	0.816	0.450	0.070	2.262	1.106	7.411
Protocol-SCCLG-ALL-2016	−0.410	0.439	0.351	0.664	0.277	1.560
Diagnosis-T-ALL	0.237	0.563	0.673	1.268	0.418	3.838
Risk-HR	2.339	1.472	0.112	10.375	0.273	136.517
Risk-IR	−1.934	1.409	0.170	0.145	0.004	1.584
rs56103835TC	−0.454	0.309	0.141	0.635	0.346	1.166
Step 2						
PLT	−0.005	0.001	0.000***	0.995	0.993	0.997
TP	−0.085	0.020	0.000***	0.918	0.883	0.954

WBC, white blood cell; PLT, platelet; ALT, alanine aminotransferase; TBIL, total bilirubin; TP, total protein; C48h, the MTX concentration of 48 h after the start of the infusion; T-ALL, T cell acute lymphoblastic leukemia; HR, high risk; IR, intermediate risk; OR, odds ratio; 95% CI, confidential intervals.

^a^ ALT ratio, ALT/upper limit of reference range.

*** *P* < 0.001.

### 3.5. Effects of rs56103835 in miR-323b and rs2114358 in miRNA secondary structure prediction

**Figure 1:**
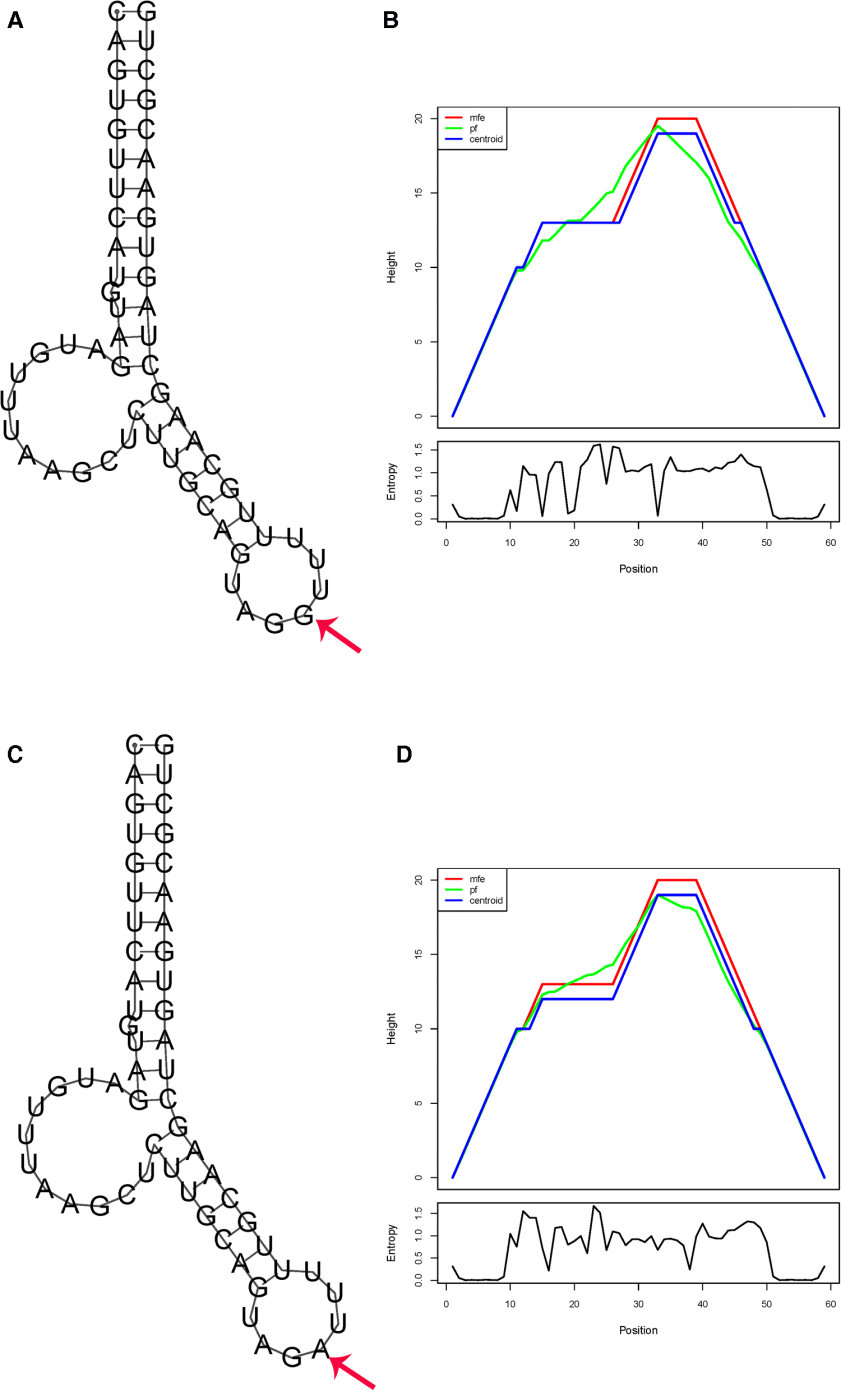
*In silico* prediction of folding structures induced by rs2114358 G>A in pre-miR-1206 secondary structure of pre-miR-1206 with rs2114356 allele G (**A**) or allele A (**C**); the mountain plot of pre-miR-1206 with rs2114356 allele G (**B**) or allele A (**D**). The free energy of the thermodynamic ensemble for pre-miR-1206 with rs2114356 alleles G and A is −19.50 and −19.30 kcal/mol, respectively.

The complete RNA sequence of has-pre-miR-1206 (Accession: NR_031611.1) and has-pre-miR-323b (Accession: NR_036133.1) was used to explore the impact of polymorphism on the RNA structure. For miR-1206 rs2114358 G>A, the free energy of the thermodynamic ensemble for G and A are −19.50 and −19.30 kcal/mol, respectively, resulting in a slight energy change (ΔΔ*G* = 0.2 kcal/mol). This change also results in a modification in the secondary structure (Figure 1).

**Figure 2:**
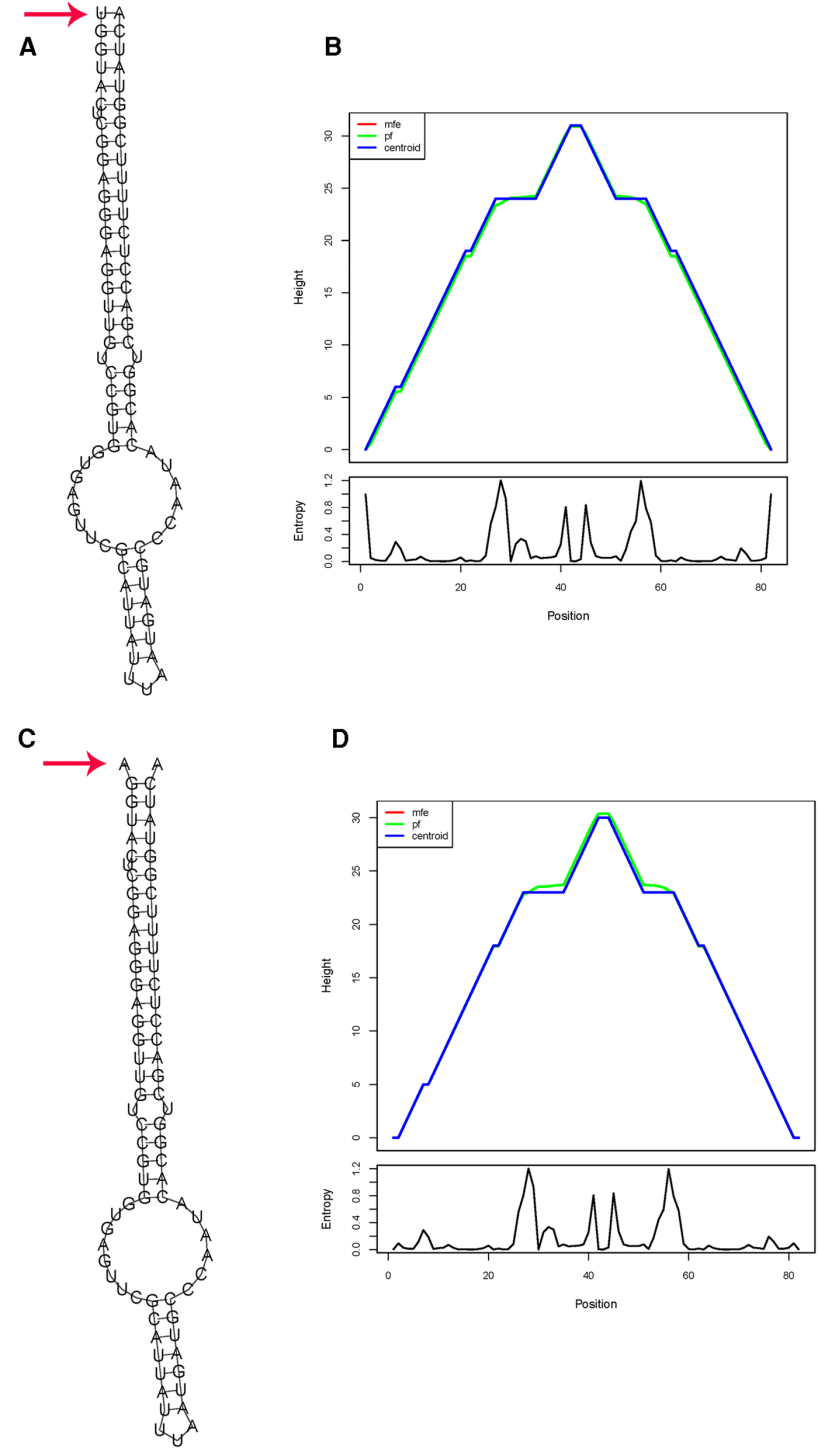
*In silico* prediction of folding structures induced by rs56103835 T>C in pre-miR-323b secondary structure of pre-miR-323b with rs56103835 allele T (**A**) or allele C (**B**); the mountain plot of pre-miR-323b with rs2114356 allele T (**C**) or allele C (**D**). The free energy of the thermodynamic ensemble for pre-miR-323b rs56103835 alleles T and C is −41.09 to −40.61 kcal/mol, respectively.

For rs56103835 T>C in miR-323b, the substitution of the T allele for a C allele induced a slight energy change of 0.48 kcal/mol (from −41.09 to −40.61 kcal/mol) (Figure 2).

### 3.6. miR-323b and miR-1206 target prediction

After target prediction analysis for miR-323b and miR-1206 was performed by using TargetScan, we found that miR-323b targeted a total of three pharmacokinetic genes involved in MTX pathways (*ABCC1*, *ABCC2*, and *ABCC4*) and one gene of the pharmacodynamic pathway (SHMT1) as shown in Table 8. miR-1206 was predicted to target three transporter genes (*SLCO1A2*, *ABCC2*, and *ABCG2*) and two enzyme genes (*TYMS* and *FPGS*) involved in MTX pharmacokinetics and pharmacodynamics (Table 8).

**Table 8:** The predicted targets for the polymorphic miRNAs.

SNP	miRNA	Predicted targets
rs2114358	miR-1206	*SLCO1A2*, *ABCC2*, *ABCG2*, *TYMS*, *FPGS*
rs56103835	miR-323b	*ABCC1*, *ABCC2*, *ABCC4*, *SHTM1*

SNP, single-nucleotide polymorphism; miRNA, microRNA.

## 4. Discussion

In this study, we explored the role of miRNA polymorphism in the HD-MTX-related hematological toxicities of pediatric patients with ALL. Our results demonstrated that after multivariable analysis, SNPs rs2114358 in miR-1206 and rs56103835 in pre-miR-323b are associated with grade 3/4 leukopenia and grade 3/4 anemia, respectively. None of the variants explored influenced thrombocytopenia.

SNP rs2114358 is located in the miR-1206 precursor. Previous studies demonstrated that the rs2114358 G-allele could significantly upregulate mature miR-1206 expression levels in colon cancer cell lines (HCT116, SW48, and KM12) and was related to the response to capecitabine-based treatment in patients with advanced colon cancer (29). The rs2114358 G-allele was associated with a higher risk of chronic lymphocytic leukemia and chronic myeloid leukemia probably by altering the secondary structure and maturation of miR-1206 (30, 31). SNP rs2114358 in miR-1206 has been reported to be involved in MTX-related mucositis during the consolidation phase; the miR-1206 rs2114358 GG genotype was associated with an increased likelihood of developing HD-MTX-related oral mucositis (17, 25). However, in the induction phase, SNP rs2114358 did not show a significant effect on oral mucositis (19). However, in our study, compared to AA and GA, patients with the rs2114356 GG genotype have a lower risk for HD-MTX-related grade 3/4 leukopenia in pediatric ALL patients (GA + AA vs. GG, OR: 2.308, 95% CI: 1.219–4372, *P* = 0.010).

There are several possible explanations for this discrepancy. First, different toxicity criteria were used. In the above two studies about oral mucositis, the condition was evaluated only once during the consolidation phase as a whole; however, in this study, leukopenia was evaluated every MTX treatment course. Second, the incidence rate of grade 3/4 leukopenia in our study was higher than that of grade 3/4 oral mucositis. In our study, 172 out of 181 patients experienced grade 3/4 leukopenia at least one time. In their study, the occurrence rate of oral mucositis was 18.8% (22/117). Lastly, the distribution of miR-1206 targets was probably different in the oral mucosa and hematopoietic cells, resulting in the different effect of rs2114358 on oral mucositis and leukopenia. Overall, the role of rs2114358 in MTX-related toxicities needs further investigation and validation.

Our *in silico* analysis showed that the allelic change from G to A in pre-miR-1206 rs2114358 induces a positive energy change (ΔΔ*G* = 0.2 kcal/mol), which is similar to that of the previous report (25). It has been suggested that positive energy changes transform the miRNA hairpin from stable to unstable status, and a decreased structure stability may reduce the mature miRNA level (32). The energy increase of GA and AA conformations results in the instability of the hairpin structure. The instability of the hairpin structure may lead to a decrease in miR-1206 expression levels and an increase in the expression levels of target genes regulated by miRNA.

With regard to miR-1206, based on bioinformatics predictions, *SLCO1A2*, *ABCC2*, *ABCG2*, *TYMS*, and *FPGS* are genes potentially associated with MTX blood concentration and toxicity. These target genes were reported to be involved in MTX toxicities (33). Among them, *SLCO1A2*, *ABCC2*, and *ABCG2* are transporters that affect the blood concentration of MTX and, thus, its toxicity (34). *TYMS* and *FPGS* are genes encoding enzymes that can also influence the blood concentration and/or toxicity of MTX (35).

SLCO1A2 actively transports MTX into the cell, while ABCG2 and ABCC2 transport MTX and its metabolites out of the cell (34). Therefore, when the expression level of miR-1206 changes, its impact on the blood concentration of MTX and its toxicity cannot be ruled out. TYMS is a crucial enzyme that catalyzes the conversion of deoxyuridine monophosphate to deoxythymidine monophosphate, and TYMS may affect both the blood concentration and the toxicity of MTX (36, 37). Previous studies have shown an inverse relationship between TYMS expression levels and MTX toxicity, indicating that higher TYMS levels are associated with lower MTX toxicity (38, 39) and higher TYMS levels are associated with a decreased risk of leukopenia. Folylpolyglutamate synthetase (FPGS) is an enzyme that converts MTX into its polyglutamate form, known as folylpolyglutamate (MTXPGs). When the level of FPGS increases, MTXPGs also increase, and the increased MTXPGs are associated with increased MTX toxicity (40, 41).

The impact of the rs2114356 G>A mutation on the risk of leukopenia cannot be inferred, and specific research is needed to determine it. According to our results, patients with the rs2114358 GA and AA genotypes have a higher risk of leukopenia, while those with the GG genotype have a lower risk.

The SNP rs56103835 was located in the pre-hsa-miR-323b-5p (also named miR-453). Rs56103835 T>C was reported to be associated with adverse event vomiting and MTX plasma levels (17). Rs56103835 was associated with the 72 h MTX level (18). In our study, the rs56103835 C allele in pre-miR-323b is associated with a higher risk of anemia. Furthermore, we analyzed the relationship between rs56103835 and 48 h MTX plasma level (C48h). The result demonstrated that C48h in the CC group is higher than that in the TC + TT group (mean ± SD: 0.404 ± 0.560 vs. 0.336 ± 0.356, *P* = 0.064), although the difference did not reach statistical significance.

For miR-323b, *in silico* analysis indicates that the substitution of the C allele for a T allele results in a slightly positive energy change (ΔΔ*G* = 0.5 kcal/mol) and thus could influence miRNA biogenesis and level of miR-323b. Positive energy changes transform the miRNA hairpin from stable to unstable status and may result in a decreased mature level (32). It has been reported that the expression level of miR-323b was slightly upregulated in tissues in the rs56103835 TC genotype compared with that in the CC genotype *in vitro* and *in vivo* (*P* = 0.014) (42). miR-323b was predicted to target genes *ABCC1*, *ABCC2*, *ABCC4*, and *SHMT1* through TargetScan; these genes are involved in MTX transport and metabolism (33). Therefore, if miR-323b-5p is changed, it would influence the expression of ABCC1, ABCC2, ABCC4, and SHMT1, and the higher risk of anima observed could probably be explained.

This study has some limitations to be addressed. First, the number of SNPs included in the study is still limited. Second, for the same patient, the hematological toxicities in different courses of MTX treatment are not independent; it may be more appropriate to construct a mixed effect model by using a kind of a “score.” In our study, we used *P* < 0.01 as the significance threshold for inclusion in the multivariable analysis to address the limitation of not using a mixed model. Finally, the predicted effects of the studied SNPs for the stability and expression level of mature miRNAs, as well as the effects on the expression levels of their target genes, require functional validation.

In conclusion, we discovered the association of the miRNA-related SNPs with the hematological toxicities of HD-MTX treatment in pediatric ALL patients. Although further functional studies and larger studies are still needed to validate the result, it might be helpful for the clinical practice of HD-MTX treatment.

## Data Availability

The original contributions presented in the study are included in the article/**Supplementary material**, further inquiries can be directed to the corresponding authors.

## References

[1] Alqarni AM Zeidler MP. How does methotrexate work? Biochem Soc Trans. (2020) 48:559–67. 10.1042/bst20190803 32239204

[2] Bedoui Y Guillot X Sélambarom J Guiraud P Giry C Jaffar-Bandjee MC Methotrexate an old drug with new tricks. Int J Mol Sci. (2019) 20:1–32. 10.3390/ijms20205023 PMC6834162 31658782

[3] Howard SC McCormick J Pui CH Buddington RK Harvey RD. Preventing and managing toxicities of high-dose methotrexate. Oncologist. (2016) 21:1471–82. 10.1634/theoncologist.2015-0164 27496039 PMC5153332

[4] Matherly LH Hou Z Deng Y. Human reduced folate carrier: translation of basic biology to cancer etiology and therapy. Cancer Metastasis Rev. (2007) 26:111–28. 10.1007/s10555-007-9046-2 17334909

[5] Meyers PA Flombaum C. High-dose methotrexate-induced renal dysfunction: is glucarpidase necessary for rescue? J Clin Oncol. (2011) 29:e180. 10.1200/jco.2010.32.8245 21220601

[6] Dhingra H Kalra M Mahajan A. Safe administration of high-dose methotrexate with minimal drug level monitoring: experience from a center in north India. Pediatr Blood Cancer. (2020) 67:e28394. 10.1002/pbc.28394 32813334

[7] Gong Y Luo L Wang L Chen J Chen F Ma Y Association of MTHFR and ABCB1 polymorphisms with MTX-induced mucositis in Chinese paediatric patients with acute lymphoblastic leukaemia, lymphoma or osteosarcoma-a retrospective cohort study. J Clin Pharm Ther. (2021) 46:1557–63. 10.1111/jcpt.13505 34346513

[8] Nearing JT Connors J Whitehouse S Van Limbergen J Macdonald T Kulkarni K Infectious complications are associated with alterations in the gut microbiome in pediatric patients with acute lymphoblastic leukemia. Front Cell Infect Microbiol. (2019) 9:28. 10.3389/fcimb.2019.00028 30838178 PMC6389711

[9] Bartel DP. MicroRNAs: genomics, biogenesis, mechanism, and function. Cell. (2004) 116:281–97. 10.1016/s0092-8674(04)00045-5 14744438

[10] Cullen BR. Transcription and processing of human microRNA precursors. Mol Cell. (2004) 16:861–5. 10.1016/j.molcel.2004.12.002 15610730

[11] Graves P Zeng Y. Biogenesis of mammalian microRNAs: a global view. Genom Proteom Bioinform. (2012) 10:239–45. 10.1016/j.gpb.2012.06.004 PMC5054211 23200133

[12] de Jonge R Hooijberg JH van Zelst BD Jansen G van Zantwijk CH Kaspers GJ Effect of polymorphisms in folate-related genes on in vitro methotrexate sensitivity in pediatric acute lymphoblastic leukemia. Blood. (2005) 106:717–20. 10.1182/blood-2004-12-4941 15797993

[13] Cammaerts S Strazisar M De Rijk P Del Favero J. Genetic variants in microRNA genes: impact on microRNA expression, function, and disease. Front Genet. (2015) 6:186. 10.3389/fgene.2015.00186 26052338 PMC4439572

[14] Fernandez N Cordiner RA Young RS Hug N Macias S Cáceres JF. Genetic variation and RNA structure regulate microRNA biogenesis. Nat Commun. (2017) 8:15114. 10.1038/ncomms15114 28466845 PMC5418625

[15] Lampropoulou DI Aravantinos G Laschos K Theodosopoulos T Papadimitriou C Gazouli M. miR-218 and miR-100 polymorphisms as markers of irinotecan-based chemotherapy response in metastatic colorectal cancer. Int J Colorectal Dis. (2019) 34:1871–7. 10.1007/s00384-019-03401-3 31598748

[16] Ryan BM Robles AI Harris CC. Genetic variation in microRNA networks: the implications for cancer research. Nat Rev Cancer. (2010) 10:389–402. 10.1038/nrc2867 20495573 PMC2950312

[17] López-López E Gutiérrez-Camino Á Piñán M Sánchez-Toledo J Uriz JJ Ballesteros J Pharmacogenetics of microRNAs and microRNAs biogenesis machinery in pediatric acute lymphoblastic leukemia. PLoS One. (2014) 9:e91261. 10.1371/journal.pone.0091261 24614921 PMC3948785

[18] Iparraguirre L Gutierrez-Camino A Umerez M Martin-Guerrero I Astigarraga I Navajas A miR-pharmacogenetics of methotrexate in childhood B-cell acute lymphoblastic leukemia. Pharmacogenet Genom. (2016) 26:517–25. 10.1097/fpc.0000000000000245 27649261

[19] Gutierrez-Camino Á Umerez M Lopez-Lopez E Santos-Zorrozua B Martin-Guerrero I de Andoin NG Involvement of miRNA polymorphism in mucositis development in childhood acute lymphoblastic leukemia treatment. Pharmacogenomics. (2018) 19:1403–12. 10.2217/pgs-2018-0113 30479191

[20] Wang SM Sun LL Zeng WX Wu WS Zhang GL. Effects of a microRNA binding site polymorphism in SLC19A1 on methotrexate concentrations in Chinese children with acute lymphoblastic leukemia. Med Oncol. (2014) 31:62. 10.1007/s12032-014-0062-0 24927955

[21] Wang SM Zeng WX Wu WS Sun LL Yan D. Association between MTHFR microRNA binding site polymorphisms and methotrexate concentrations in Chinese pediatric patients with acute lymphoblastic leukemia. J Gene Med. (2017) 19:353–9. 10.1002/jgm.2990 28990296

[22] Wang SM Zeng WX Wu WS Sun LL Yan D. Association between a microRNA binding site polymorphism in SLCO1A2 and the risk of delayed methotrexate elimination in Chinese children with acute lymphoblastic leukemia. Leuk Res. (2018) 65:61–6. 10.1016/j.leukres.2018.01.004 29306656

[23] Wang XQ Li Y Su X Zhang L Liu CM Liu H Haplotype-based association of two SNPs in miR-323b with unexplained recurrent spontaneous abortion in a Chinese Han population. J Cell Physiol. (2018) 233:6001–17. 10.1002/jcp.26415 29271476

[24] Li XY Li JQ Luo XQ Wu XD Sun X Xu HG Reduced intensity of early intensification does not increase the risk of relapse in children with standard risk acute lymphoblastic leukemia—a multi-centric clinical study of GD-2008-ALL protocol. BMC Cancer. (2021) 21:59. 10.1186/s12885-020-07752-x 33435902 PMC7805214

[25] Gutierrez-Camino A Oosterom N den Hoed MAH Lopez-Lopez E Martin-Guerrero I Pluijm SMF The miR-1206 microRNA variant is associated with methotrexate-induced oral mucositis in pediatric acute lymphoblastic leukemia. Pharmacogenet Genom. (2017) 27:303–6. 10.1097/fpc.0000000000000291 28628559

[26] Gutierrez-Camino A Martin-Guerrero I Dolzan V Jazbec J Carbone-Bañeres A Garcia de Andoin N Involvement of SNPs in miR-3117 and miR-3689d2 in childhood acute lymphoblastic leukemia risk. Oncotarget. (2018) 9:22907–14. 10.18632/oncotarget.25144 29796161 PMC5955428

[27] Li MJ Chang HH Yang YL Lu MY Shao PL Fu CM Infectious complications in children with acute lymphoblastic leukemia treated with the Taiwan Pediatric Oncology Group protocol: a 16-year tertiary single-institution experience. Pediatr Blood Cancer. (2017) 64:1–9. 10.1002/pbc.26535 28371256

[28] Gruber AR Lorenz R Bernhart SH Neuböck R Hofacker IL. The Vienna RNA websuite. Nucleic Acids Res. (2008) 36:W70–74. 10.1093/nar/gkn188 18424795 PMC2447809

[29] Kim HK Prokunina-Olsson L Chanock SJ. Common genetic variants in miR-1206 (8q24.2) and miR-612 (11q13.3) affect biogenesis of mature miRNA forms. PLoS One. (2012) 7:e47454. 10.1371/journal.pone.0047454 23077621 PMC3471815

[30] Hassan FM. The association of rs2114358 in the miR-1206 polymorphism to chronic myeloid leukemia. Microrna. (2019) 8:248–52. 10.2174/2211536608666190102143439 30605069

[31] Martin-Guerrero I Gutierrez-Camino A Lopez-Lopez E Bilbao-Aldaiturriaga N Pombar-Gomez M Ardanaz M Genetic variants in miRNA processing genes and pre-miRNAs are associated with the risk of chronic lymphocytic leukemia. PLoS One. (2015) 10:e0118905. 10.1371/journal.pone.0118905 25793711 PMC4368096

[32] Gong J Tong Y Zhang HM Wang K Hu T Shan G Genome-wide identification of SNPs in microRNA genes and the SNP effects on microRNA target binding and biogenesis. Hum Mutat. (2012) 33:254–63. 10.1002/humu.21641 22045659

[33] Hu YH Zhou L Wang SS Jing X Guo HL Sun F Methotrexate disposition in pediatric patients with acute lymphoblastic leukemia: what have we learnt from the genetic variants of drug transporters. Curr Pharm Des. (2019) 25:627–34. 10.2174/1381612825666190329141003 30931851

[34] Mikkelsen TS Thorn CF Yang JJ Ulrich CM French D Zaza G PharmGKB summary: methotrexate pathway. Pharmacogenet Genomics. (2011) 21:679–86. 10.1097/FPC.0b013e328343dd93 21317831 PMC3139712

[35] Hashiguchi M Shimizu M Hakamata J Tsuru T Tanaka T Suzaki M Genetic polymorphisms of enzyme proteins and transporters related to methotrexate response and pharmacokinetics in a Japanese population. J Pharm Health Care Sci. (2016) 2:35. 10.1186/s40780-016-0069-0 27980801 PMC5148839

[36] Chaabane S Messedi M Akrout R Ben Hamad M Turki M Marzouk S Association of hyperhomocysteinemia with genetic variants in key enzymes of homocysteine metabolism and methotrexate toxicity in rheumatoid arthritis patients. Inflamm Res. (2018) 67:703–10. 10.1007/s00011-018-1161-8 29796841

[37] DiPaolo A Chu E. The role of thymidylate synthase as a molecular biomarker. Clin Cancer Res. (2004) 10:411–2. 10.1158/1078-0432.ccr-1198-03 14760058

[38] Faganel Kotnik B Grabnar I Bohanec Grabar P Dolžan V Jazbec J. Association of genetic polymorphism in the folate metabolic pathway with methotrexate pharmacokinetics and toxicity in childhood acute lymphoblastic leukaemia and malignant lymphoma. Eur J Clin Pharmacol. (2011) 67:993–1006. 10.1007/s00228-011-1046-z 21509569

[39] Oosterom N Berrevoets M den Hoed MAH Zolk O Hoerning S Pluijm SMF The role of genetic polymorphisms in the thymidylate synthase (TYMS) gene in methotrexate-induced oral mucositis in children with acute lymphoblastic leukemia. Pharmacogenet Genomics. (2018) 28:223–9. 10.1097/fpc.0000000000000352 30222710

[40] Liu SG Li ZG Cui L Gao C Li WJ Zhao XX. Effects of methylenetetrahydrofolate reductase gene polymorphisms on toxicities during consolidation therapy in pediatric acute lymphoblastic leukemia in a Chinese population. Leuk Lymphoma. (2011) 52:1030–40. 10.3109/10428194.2011.563883 21534867

[41] Wang SM Sun LL Zeng WX Wu WS Zhang GL. Influence of genetic polymorphisms of FPGS, GGH, and MTHFR on serum methotrexate levels in Chinese children with acute lymphoblastic leukemia. Cancer Chemother Pharmacol. (2014) 74:283–9. 10.1007/s00280-014-2507-8 24908438

[42] Yu SJ Kim JW Lee JH Yoon JH Lee HS Cheong JY Association of a microRNA-323b polymorphism with the persistence of hepatitis B virus infection by the enhancement of viral replication. J Viral Hepat. (2014) 21:853–9. 10.1111/jvh.12215 24341744

