# Supplementary material for: Impact of microRNA polymorphisms on high-dose methotrexate-related hematological toxicities in pediatric acute lymphoblastic leukemia
Source: Front Pediatr. 2023 Jun 13;11:1153767. doi: 10.3389/fped.2023.1153767 (PMC10293614; doi:10.3389/fped.2023.1153767)
Supplement: Supplementary file 1 [file Table1.docx]

Table S1 Genes and SNPs included in our study

| SNP | Gene | Allele | Chromosome | Location^b^ | Functional Consequence ^a^ | HWE(p-value) |
| --- | --- | --- | --- | --- | --- | --- |
| rs10505168 | hsa-miR-2053 | A>G | 8 | 112643523 | pre-miRNA | 0.273 |
| rs1572687 | hsa-mir-5007 | C>T | 13 | 55174538 | pre-miRNA | 0.129 |
| [rs2114358](https://www.ncbi.nlm.nih.gov/snp/rs2114358) | hsa-mir-1206 | T>C | 8 | 128008933 | pre-miRNA | 0.263 |
| rs2368392 | hsa-mir-604 | C>T | 10 | 29545074 | pre-miRNA | 0.941 |
| rs243080 | hsa-mir-4432 | C>T | 2 | 60387437 | pre-miRNA | 0.604 |
| rs35613341 | hsa-mir-5189 | C>G | 16 | 88468999 | mature | 0.696 |
| rs4674470 | hsa-mir-4268 | T>C | 2 | 219906502 | pre-miRNA | 0.687 |
| rs4909237 | hsa-mir-595 | C>T | 7 | 158532811 | pre-miRNA | 0.756 |
| rs56103835 | hsa-mir-323b | T>C | 14 | 101056219 | pre-miRNA | 0.604 |
| rs56292801 | hsa-mir-5189 | G>A | 16 | 88468933 | pre-miRNA | 0.579 |
| rs60871950 | hsa-mir-4467 | G>A | 7 | 102471489 | mature | 0.940 |
| rs62571442 | hsa-miR-3689 | A>G | 9 | 134850278 | pre-miRNA | 0.693 |
| rs78790512 | hsa-mir-6083 | G>A | 3 | 124374373 | pre-miRNA | 0.761 |
| rs1055070 | hsa-miR-4700 | T>G | 12 | 120723245 | mature | - |
| rs8078913 | hsa-miR-4520-2; hsa-miR-4520-1 | C>A | 17 | 6655449 | mature | - |

SNP: Single nucleotide polymorphism

a <http://bioinfo.life.hust.edu.cn/miRNASNP/#!/>

b https://www.ncbi.nlm.nih.gov/snp/?term=
