# Supplementary material for: Impact of microRNA polymorphisms on high-dose methotrexate-related hematological toxicities in pediatric acute lymphoblastic leukemia
Source: Front Pediatr. 2023 Jun 13;11:1153767. doi: 10.3389/fped.2023.1153767 (PMC10293614; doi:10.3389/fped.2023.1153767)
Supplement: Supplementary file 2 [file Table2.docx]

Table S2 The association between SNPs and leukopenia

| SNP | Genotype | Grade0 | Grade1 | Grade2 | Grade3 | Grade4 | p-value^a^ | P_fdr_^b^ |
| --- | --- | --- | --- | --- | --- | --- | --- | --- |
| rs10505168 | C | 1 | 3 | 27 | 38 | 73 | 0.068 | 0.295 |
|  | CT | 10 | 29 | 46 | 75 | 142 |  |  |
|  | T | 2 | 15 | 35 | 59 | 99 |  |  |
| rs1572687 | C | 9 | 33 | 69 | 93 | 190 | 0.344 | 0.603 |
|  | CT | 4 | 10 | 35 | 65 | 98 |  |  |
|  | T | 0 | 4 | 4 | 14 | 26 |  |  |
| rs2114358 | A | 1 | 18 | 53 | 73 | 144 | 0.000 | 0.000 |
|  | G | 4 | 9 | 12 | 17 | 13 |  |  |
|  | GA | 8 | 20 | 43 | 82 | 157 |  |  |
| rs2368392 | A | 1 | 5 | 15 | 14 | 37 | 0.156 | 0.457 |
|  | G | 6 | 19 | 48 | 63 | 150 |  |  |
|  | GA | 6 | 23 | 45 | 95 | 127 |  |  |
| rs243080 | A | 5 | 17 | 48 | 69 | 144 | 0.890 | 0.890 |
|  | G | 1 | 7 | 10 | 20 | 33 |  |  |
|  | GA | 7 | 23 | 50 | 83 | 137 |  |  |
| rs35613341 | C | 5 | 10 | 23 | 37 | 58 | 0.458 | 0.603 |
|  | CG | 5 | 28 | 49 | 80 | 150 |  |  |
|  | G | 3 | 9 | 36 | 55 | 106 |  |  |
| rs4674470 | C | 1 | 4 | 10 | 14 | 21 | 0.211 | 0.457 |
|  | CT | 2 | 26 | 45 | 74 | 124 |  |  |
|  | T | 10 | 17 | 53 | 84 | 169 |  |  |
| rs4909237 | C | 10 | 27 | 57 | 96 | 203 | 0.181 | 0.457 |
|  | CT | 3 | 19 | 47 | 64 | 101 |  |  |
|  | T | 0 | 1 | 4 | 12 | 10 |  |  |
| rs56103835 | C | 3 | 20 | 44 | 69 | 134 | 0.633 | 0.686 |
|  | T | 0 | 5 | 11 | 25 | 34 |  |  |
|  | TC | 10 | 22 | 53 | 78 | 146 |  |  |
| rs56292801 | A | 3 | 9 | 36 | 55 | 106 | 0.510 | 0.603 |
|  | G | 5 | 10 | 23 | 37 | 61 |  |  |
|  | GA | 5 | 28 | 49 | 80 | 147 |  |  |
| rs60871950 | A | 0 | 3 | 7 | 8 | 8 | 0.011 | 0.072 |
|  | AG | 1 | 15 | 45 | 42 | 112 |  |  |
|  | G | 12 | 29 | 56 | 122 | 194 |  |  |
| rs62571442 | A | 0 | 2 | 3 | 5 | 15 | 0.464 | 0.603 |
|  | G | 6 | 27 | 74 | 118 | 211 |  |  |
|  | GA | 7 | 18 | 31 | 49 | 88 |  |  |
| rs78790512 | AG | 1 | 3 | 8 | 5 | 12 | 0.376 | 0.603 |
|  | G | 12 | 44 | 100 | 167 | 302 |  |  |

SNP: single nucleotide polymorphism

a: Fisher's exact test

b: Benjamini-Hochberg

Table S3 The Association between SNPs with HD-MTX related anemia

| SNP | Genotype | Grade0 | Grade1 | Grade2 | Grade3 | Grade4 | p-value^a^ | P_fdr_^b^ |
| --- | --- | --- | --- | --- | --- | --- | --- | --- |
| rs10505168 | C | 0 | 47 | 48 | 44 | 3 | 0.001 | 0.007 |
|  | CT | 17 | 119 | 100 | 63 | 3 |  |  |
|  | T | 6 | 60 | 84 | 55 | 5 |  |  |
| rs1572687 | C | 17 | 144 | 129 | 97 | 7 | 0.702 | 0.702 |
|  | CT | 5 | 67 | 83 | 54 | 3 |  |  |
|  | T | 1 | 15 | 20 | 11 | 1 |  |  |
| rs2114358 | A | 8 | 107 | 97 | 70 | 7 | 0.416 | 0.486 |
|  | G | 4 | 20 | 20 | 10 | 1 |  |  |
|  | GA | 11 | 99 | 115 | 82 | 3 |  |  |
| rs2368392 | A | 0 | 32 | 18 | 20 | 2 | 0.015 | 0.0498 |
|  | G | 15 | 85 | 99 | 82 | 5 |  |  |
|  | GA | 8 | 109 | 115 | 60 | 4 |  |  |
| rs243080 | A | 6 | 88 | 111 | 72 | 6 | 0.137 | 0.254 |
|  | G | 4 | 31 | 24 | 11 | 1 |  |  |
|  | GA | 13 | 107 | 97 | 79 | 4 |  |  |
| rs35613341 | C | 10 | 43 | 41 | 38 | 1 | 0.021 | 0.0498 |
|  | CG | 4 | 108 | 111 | 84 | 5 |  |  |
|  | G | 9 | 75 | 80 | 40 | 5 |  |  |
| rs4674470 | C | 2 | 19 | 14 | 15 | 0 | 0.226 | 0.335 |
|  | CT | 13 | 81 | 105 | 69 | 3 |  |  |
|  | T | 8 | 126 | 113 | 78 | 8 |  |  |
| rs4909237 | C | 11 | 131 | 151 | 94 | 6 | 0.449 | 0.486 |
|  | CT | 12 | 85 | 73 | 60 | 4 |  |  |
|  | T | 0 | 10 | 8 | 8 | 1 |  |  |
| rs56103835 | C | 10 | 71 | 90 | 91 | 8 | 0.000 | 0.000 |
|  | T | 5 | 30 | 27 | 13 | 0 |  |  |
|  | TC | 8 | 125 | 115 | 58 | 3 |  |  |
| rs56292801 | A | 9 | 75 | 80 | 40 | 5 | 0.017 | 0.0498 |
|  | G | 10 | 43 | 42 | 40 | 1 |  |  |
|  | GA | 4 | 108 | 110 | 82 | 5 |  |  |
| rs60871950 | A | 2 | 11 | 10 | 3 | 0 | 0.263 | 0.342 |
|  | AG | 4 | 82 | 69 | 55 | 5 |  |  |
|  | G | 17 | 133 | 153 | 104 | 6 |  |  |
| rs62571442 | A | 0 | 7 | 4 | 13 | 1 | 0.023 | 0.0498 |
|  | G | 18 | 152 | 147 | 112 | 7 |  |  |
|  | GA | 5 | 67 | 81 | 37 | 3 |  |  |
| rs78790512 | AG | 1 | 16 | 7 | 5 | 0 | 0.232 | 0.335 |
|  | G | 22 | 210 | 225 | 157 | 11 |  |  |

SNP: single nucleotide polymorphism

a: Fisher's exact test

b: Benjamini-Hochberg

Table S4 The association between SNPs and thrombocytopenia

| SNP | Genotype | Grade0 | Grade1 | Grade2 | Grade3 | Grade4 | p-value ^a^ | P_fdr_^b^ |
| --- | --- | --- | --- | --- | --- | --- | --- | --- |
| rs10505168 | C | 96 | 12 | 6 | 10 | 18 | 0.090 | 0.195 |
|  | CT | 216 | 21 | 15 | 17 | 33 |  |  |
|  | T | 148 | 4 | 8 | 14 | 36 |  |  |
| rs1572687 | C | 278 | 18 | 19 | 30 | 49 | 0.031 | 0.081 |
|  | CT | 142 | 19 | 10 | 11 | 30 |  |  |
|  | T | 40 | 0 | 0 | 0 | 8 |  |  |
| rs2114358 | A | 200 | 17 | 12 | 17 | 43 | 0.504 | 0.596 |
|  | G | 47 | 2 | 1 | 1 | 4 |  |  |
|  | GA | 213 | 18 | 16 | 23 | 40 |  |  |
| rs2368392 | A | 49 | 4 | 4 | 5 | 10 | 0.211 | 0.305 |
|  | G | 189 | 14 | 15 | 19 | 49 |  |  |
|  | GA | 222 | 19 | 10 | 17 | 28 |  |  |
| rs243080 | A | 197 | 14 | 13 | 19 | 40 | 0.418 | 0.543 |
|  | G | 57 | 1 | 1 | 2 | 10 |  |  |
|  | GA | 206 | 22 | 15 | 20 | 37 |  |  |
| rs35613341 | C | 93 | 4 | 7 | 6 | 23 | 0.023 | 0.075 |
|  | CG | 204 | 21 | 19 | 25 | 43 |  |  |
|  | G | 163 | 12 | 3 | 10 | 21 |  |  |
| rs4674470 | C | 32 | 5 | 4 | 3 | 6 | 0.146 | 0.271 |
|  | CT | 180 | 20 | 14 | 20 | 37 |  |  |
|  | T | 248 | 12 | 11 | 18 | 44 |  |  |
| rs4909237 | C | 276 | 26 | 17 | 27 | 47 | 0.669 | 0.669 |
|  | CT | 161 | 11 | 12 | 13 | 37 |  |  |
|  | T | 23 | 0 | 0 | 1 | 3 |  |  |
| rs56103835 | C | 162 | 16 | 15 | 22 | 55 | 0.000 | 0.000 |
|  | T | 59 | 6 | 2 | 4 | 4 |  |  |
|  | TC | 239 | 15 | 12 | 15 | 28 |  |  |
| rs56292801 | A | 163 | 12 | 3 | 10 | 21 | 0.021 | 0.075 |
|  | G | 95 | 4 | 8 | 6 | 23 |  |  |
|  | GA | 202 | 21 | 18 | 25 | 43 |  |  |
| rs60871950 | A | 22 | 3 | 1 | 0 | 0 | 0.014 | 0.075 |
|  | AG | 151 | 8 | 4 | 18 | 34 |  |  |
|  | G | 287 | 26 | 24 | 23 | 53 |  |  |
| rs62571442 | A | 14 | 1 | 1 | 2 | 7 | 0.199 | 0.305 |
|  | G | 303 | 24 | 19 | 26 | 64 |  |  |
|  | GA | 143 | 12 | 9 | 13 | 16 |  |  |
| rs78790512 | AG | 25 | 1 | 0 | 1 | 2 | 0.622 | 0.669 |
|  | G | 435 | 36 | 29 | 40 | 85 |  |  |

SNP: single nucleotide polymorphism

a: Fisher's exact test

b: Benjamini-Hochberg
