# Supplementary material for: Impact of microRNA polymorphisms on high-dose methotrexate-related hematological toxicities in pediatric acute lymphoblastic leukemia
Source: Front Pediatr. 2023 Jun 13;11:1153767. doi: 10.3389/fped.2023.1153767 (PMC10293614; doi:10.3389/fped.2023.1153767)
Supplement: Supplementary file 3 [file Table5.docx]

Table S5 Correlation between clinical factors and leukopenia

| Variable | Grade 0 | Grade 1 | Grade 2 | Grade 3 | Grade 4 | P-value^c^ |
| --- | --- | --- | --- | --- | --- | --- |
| age(day) | 1757.73±929.31(460) | 2132.45±954.01(37) | 1800.13±1010.25(29) | 2070.01±1182.13(41) | 2209.28±1258.46(87) | 0.409 |
| weight(kg) | 18.55±8.15(460) | 20.96±8.08(37) | 18.77±8.6(29) | 20.51±8.65(41) | 21.14±9.6(87) | 0.708 |
| WBC(*109/L) | 3.24±1.35(460) | 2.94±2.15(37) | 3.9±3.12(29) | 3.39±3.87(41) | 3.17±1.65(87) | 0.000 |
| RBC(*1012/L) | 3.36±0.47(460) | 3.14±0.45(37) | 3.27±0.48(29) | 3.29±0.51(41) | 3.23±0.49(87) | 0.000 |
| PLT(*109/L) | 263.15±133.7(460) | 179.97±98.55(37) | 182.21±102.38(29) | 215.98±149.61(41) | 169.2±121.72(87) | 0.000 |
| ALT ratio^a^ | 1.05±0.95(460) | 1.85±1.37(37) | 1.33±0.97(29) | 1.45±0.98(41) | 1.66±1.36(87) | 0.000 |
| TBIL(μmol/L) | 7.85±3.22(460) | 9.16±4.67(37) | 9.12±3.42(29) | 9.45±4.27(41) | 10.74±6.59(87) | 0.372 |
| TP(g/L) | 63.33±4.55(460) | 60.52±5.29(37) | 60.59±5.16(29) | 59.62±5.41(41) | 61.14±6.6(87) | 0.000 |
| Cr ratio^b^ | 0.41±0.17(460) | 0.44±0.16(37) | 0.47±0.18(29) | 0.45±0.15(41) | 0.43±0.14(87) | 0.470 |
| C48h(μmol/L) | 0.28±0.13(13) | 0.4±0.49(36) | 0.29±0.19(29) | 0.55±0.72(41) | 1.2±3.8(86) | 0.079 |
| C72h(μmol/L | 0.13(1) | 0.15±0.19(25) | 0.1±0.08(19) | 0.19±0.2(27) | 0.37±0.77(58) | 0.073 |
| Sex |  |  |  |  |  |  |
| male | 5 | 25 | 58 | 93 | 194 | 0.192 |
| female | 8 | 22 | 50 | 79 | 120 |  |
| Dose |  |  |  |  |  |  |
| 2 | 9 | 32 | 51 | 52 | 53 | 0 |
| 5 | 4 | 15 | 57 | 120 | 261 |  |
| protocol |  |  |  |  |  |  |
| GD2008 | 3 | 10 | 24 | 22 | 61 | 0.255 |
| SCCLG-ALL-2016 | 10 | 37 | 84 | 150 | 253 |  |
| Type |  |  |  |  |  |  |
| B-ALL | 13 | 47 | 105 | 169 | 294 | 0.043 |
| T-ALL | 0 | 0 | 3 | 3 | 20 |  |
| Risk |  |  |  |  |  |  |
| LR | 6 | 23 | 33 | 39 | 35 | 0 |
| IR | 7 | 24 | 73 | 119 | 151 |  |
| HR | 0 | 0 | 2 | 14 | 128 |  |

Values are shown as means (n) or n where appropriate.

WBC: white blood count; RBC: red blood count; PLT: platelet; ALT: alanine aminotransferase; TBIL: total bilirubin; TP: total protein; Cr: creatinine;

C48h: the MTX concentration of 48h after the start of the infusion; C72h the MTX concentration of 72h after the start of the infusion;

B-ALL: B-cell acute lymphoblastic leukemia; T-ALL: B-cell acute lymphoblastic leukemia.

LR: low risk; IR: intermediate risk; HR: high risk.

a ALT ratio = ALT/upper limit of reference range

b Creatinine ratio = creatinine/upper limit of reference range.

c categorical variables: chisq-test or Fisher’s exact test; numeric variables: ANOVA or Mann–Whitney–Wilcoxon test
