# Supplementary material for: Impact of microRNA polymorphisms on high-dose methotrexate-related hematological toxicities in pediatric acute lymphoblastic leukemia
Source: Front Pediatr. 2023 Jun 13;11:1153767. doi: 10.3389/fped.2023.1153767 (PMC10293614; doi:10.3389/fped.2023.1153767)
Supplement: Supplementary file 4 [file Table6.docx]

Table S6 Correlation between clinical factors and anemia

| Variable | Grade 0 | Grade 1 | Grade 2 | Grade 3 | Grade 4 | p-value^c^ |
| --- | --- | --- | --- | --- | --- | --- |
| Age(day) | 2143.62±1034.34(23) | 1729.53±827.97(226) | 1822±1041.64(232) | 2008.48±1145.66(162) | 2589.47±1213.49(11) | 0.022 |
| weight(kg) | 22.14±11.57(23) | 18.4±7.65(226) | 18.8±8.27(232) | 19.93±8.93(162 | 24.87±9.73(11) | 0.017 |
| WBC(*109/L) | 3.18±1(23) | 3.45±1.43(226) | 3.02±1.43(232 | 3.17±1.93(162) | 5.45±7.13(11) | 0.000 |
| RBC(*1012/L) | 3.86±0.33(23) | 3.56±0.42(226) | 3.18±0.4(232) | 3.13±0.49(162) | 3.16±0.56(11) | 0.000 |
| PLT(*109/L) | 246.96±60.19(23) | 265.54±129.23(226) | 239.78±137.55(232) | 207.8±138.17(162) | 143.73±159.52(11) | 0.000 |
| ALT ratio ^a^ | 1.27±0.82(23) | 0.9±0.82(226) | 1.14±0.96(232 | 1.71±1.35(162) | 1.68±0.9(11) | 0.000 |
| TBIL(μmol/L) | 7.23±2.67(23) | 7.35±2.79(226) | 8.49±3.76(232) | 9.63±4.81(162) | 16.18±9.75(11) | 0.000 |
| TP(g/L) | 62.87±3.6(23) | 63.81±4.03(226) | 63.15±4.32(232) | 59.93±6.65(162) | 60.42±5.16(11) | 0.000 |
| Cr ratio ^b^ | 0.38±0.16(23) | 0.4±0.16(226) | 0.42±0.18(232 | 0.44±0.16(162) | 0.44±0.08(11) | 0.001 |
| C48h(μmol/L) | 0.24±0.17(23) | 0.3±0.31(223) | 0.39±0.5(228) | 1.07±4.36(158) | 0.61±0.73(11) | 0.025 |
| C72h(μmol/L) | 0.08±0.07(4) | 0.12±0.08(90) | 0.17±0.2(111) | 0.38±1.15(97) | 0.13±0.05(7) | 0.267 |
| Sex |  |  |  |  |  |  |
| male | 11 | 128 | 127 | 100 | 9 | 0.239 |
| female | 12 | 98 | 105 | 62 | 2 |  |
| Dose |  |  |  |  |  |  |
| 2 | 15 | 113 | 62 | 7 | 0 | 0.000 |
| 5 | 8 | 113 | 170 | 155 | 11 |  |
| protocol |  |  |  |  |  |  |
| GD2008 | 8 | 47 | 28 | 35 | 2 | 0.015 |
| SCCLG-ALL-2016 | 15 | 179 | 204 | 127 | 9 |  |
| TYPE |  |  |  |  |  |  |
| B-ALL | 23 | 225 | 222 | 149 | 9 | 0.000 |
| T-ALL | 0 | 1 | 10 | 13 | 2 |  |
| risk |  |  |  |  |  |  |
| LR | 7 | 77 | 46 | 6 | 0 | 0.000 |
| IR | 16 | 138 | 161 | 56 | 3 |  |
| HR | 0 | 11 | 25 | 100 | 8 |  |

Values are shown as means (n) or n where appropriate.

WBC: white blood count; RBC: red blood count; PLT: platelet; ALT: alanine aminotransferase; TBIL: total bilirubin; TP: total protein; Cr: creatinine;

C48h: the MTX concentration of 48h after the start of the infusion; C72h the MTX concentration of 72h after the start of the infusion;

B-ALL: B-cell acute lymphoblastic leukemia; T-ALL: B-cell acute lymphoblastic leukemia.

LR: low risk; IR: intermediate risk; HR: high risk.

a ALT ratio = ALT/upper limit of reference range

b Creatinine ratio = creatinine/upper limit of reference range.

c categorical variables: chisq-test or Fisher’s exact test; numeric variables: ANOVA or Mann–Whitney–Wilcoxon test
