# Supplementary material for: Impact of microRNA polymorphisms on high-dose methotrexate-related hematological toxicities in pediatric acute lymphoblastic leukemia
Source: Front Pediatr. 2023 Jun 13;11:1153767. doi: 10.3389/fped.2023.1153767 (PMC10293614; doi:10.3389/fped.2023.1153767)
Supplement: Supplementary file 5 [file Table7.docx]

Table S7 Correlation between clinical factors and thrombocytopenia

| variable | grade 0 | grade 1 | grade 2 | grade 3 | grade 4 | p-value^c^ |
| --- | --- | --- | --- | --- | --- | --- |
| Age(day) | 1757.73±929.31(460) | 2132.45±954.01(37) | 1800.13±1010.25(29) | 2070.01±1182.13(41) | 2209.28±1258.46(87) | 0.005 |
| Wegiht(kg) | 18.55±8.15(460) | 20.96±8.08(37) | 18.77±8.6(29) | 20.51±8.65(41) | 21.14±9.6(87) | 0.030 |
| WBC(*109/L) | 3.24±1.35(460) | 2.94±2.15(37) | 3.9±3.12(29) | 3.39±3.87(41) | 3.17±1.65(87) | 0.003 |
| RBC(*10^12^/L) | 3.36±0.47(460) | 3.14±0.45(37) | 3.27±0.48(29) | 3.29±0.51(41) | 3.23±0.49(87) | 0.019 |
| PLT(*10^9^/L) | 263.15±133.7(460) | 179.97±98.55(37) | 182.21±102.38(29) | 215.98±149.61(41) | 169.2±121.72(87) | 0.000 |
| ALT Ratio ^a^ | 1.05±0.95(460) | 1.85±1.37(37) | 1.33±0.97(29) | 1.45±0.98(41) | 1.66±1.36(87) | 0.000 |
| TBIL (μmol/L) | 7.85±3.22(460) | 9.16±4.67(37) | 9.12±3.42(29) | 9.45±4.27(41) | 10.74±6.59(87) | 0.000 |
| TP(g/L) | 63.33±4.55(460) | 60.52±5.29(37) | 60.59±5.16(29) | 59.62±5.41(41) | 61.14±6.6(87) | 0.000 |
| Cr Ratio ^b^ | 0.41±0.17(460) | 0.44±0.16(37) | 0.47±0.18(29) | 0.45±0.15(41) | 0.43±0.14(87) | 0.015 |
| C48h(μmol/L | 0.28±0.13(13) | 0.4±0.49(36) | 0.29±0.19(29) | 0.55±0.72(41) | 1.2±3.8(86) | 0.002 |
| C72h(μmol/L) | 0.13(1) | 0.15±0.19(25) | 0.1±0.08(19) | 0.19±0.2(27) | 0.37±0.77(58) | 0.066 |
| Sex |  |  |  |  |  |  |
| male | 252 | 21 | 16 | 23 | 63 | 0.052 |
| female | 208 | 16 | 13 | 18 | 24 |  |
| Dose |  |  |  |  |  |  |
| 2 | 183 | 7 | 3 | 0 | 4 | 0.000 |
| 5 | 277 | 30 | 26 | 41 | 83 |  |
| protocol |  |  |  |  |  |  |
| GD2008 | 74 | 8 | 2 | 14 | 22 | 0.007 |
| SCCLG-ALL-2016 | 386 | 29 | 27 | 27 | 65 |  |
| type |  |  |  |  |  |  |
| B-ALL | 452 | 36 | 27 | 36 | 77 | 0.000 |
| T-ALL | 8 | 1 | 2 | 5 | 10 |  |
| risk |  |  |  |  |  |  |
| LR | 125 | 4 | 3 | 0 | 4 | 0.000 |
| IR | 307 | 26 | 15 | 13 | 13 |  |
| HR | 28 | 7 | 11 | 28 | 70 |  |

Values are shown as means (n) or n where appropriate.

WBC: white blood count; RBC: red blood count; PLT: platelet; ALT: alanine aminotransferase; TBIL: total bilirubin; TP: total protein; Cr: creatinine;

C48h: the MTX concentration of 48h after the start of the infusion; C72h the MTX concentration of 72h after the start of the infusion;

B-ALL: B-cell acute lymphoblastic leukemia; T-ALL: B-cell acute lymphoblastic leukemia.

LR: low risk; IR: intermediate risk; HR: high risk.

a ALT ratio = ALT/upper limit of reference range

b Creatinine ratio = creatinine/upper limit of reference range.

c categorical variables: chisq-test or Fisher’s exact test; numeric variables: ANOVA or Mann–Whitney–Wilcoxon test
